# Potential of silver against human colon cancer: (synthesis, characterization and crystal structures of xylyl (*Ortho*, *meta*, &*Para*) linked *bis*-benzimidazolium salts and Ag(I)-NHC complexes: *In vitro* anticancer studies)

**DOI:** 10.1186/1752-153X-7-27

**Published:** 2013-02-07

**Authors:** Muhammad Adnan Iqbal, Rosenani A Haque, Siti Fatimah Nasri, AMS Abdul Majid, Mohamed B Khadeer Ahamed, Elham Farsi, Tabinda Fatima

**Affiliations:** 1 The School of Chemical Sciences, Universiti Sains Malaysia, 11800 USM, Penang, Malaysia; 2 EMAN Testing and Research Laboratories, The School of Pharmaceutical Sciences, Universiti Sains Malaysia, 11800 USM, Penang, Malaysia

## Abstract

**Background:**

Since the first successful synthesis of Ag(I)-N-heterocyclic carbene complex in 1993, this class of compounds has been extensively used for transmetallation reactions where the direct synthesis using other metal ions was either difficult or impossible. Initially, silver(I)-NHC complexes were tested for their catalytic potential but could not get fame because of lower potential compare to other competent compounds in this field; however, these compounds proved to have vital antimicrobial activities. These encouraging biomedical applications further convinced researchers to test these compounds against cancer. The current work has been carried out with this aim.

**Results:**

*N*-*i*propylbenzimidazole was synthesized by reaction of benzimidazole with ^*i*^propyl bromide. The subsequent treatment of the resulting *N*-alkylbenzimidazole with *ortho*/*meta*/*para*-(bromomethylene) benzene afforded corresponding *bis*-benzimidazolium bromides (**5**-**7**). The counter anion (Br-) of each salt was replaced by hexaflourophosphate (PF_6_^-^) for the ease of handling and further purification (**8**-**10**). Each salt (Ligand), in halide form, was further allowed to react with Ag_2_O with stirring at room temperature for a period of two days to synthesize dinuclear Ag(I)-NHC complexes (**11**-**13**). All synthesized compounds were characterized by spectroscopic techniques and microanalysis. Molecular structures of compounds **5**, **9** &**10** were established through single crystal x-ray diffraction technique. All the compounds were assessed for their anti-proliferation test on human colorectal cancer cell line (HCT 116). Results showed that the ligands (**5-10**) showed mild to negligible cytotoxicity on HCT 116 cells whereas respective silver complexes (**11-13**) exhibited dose dependent cytotoxicity towards the colon cancer cells with IC_50_ ranges between 9.7 to 44.5 μM. Interestingly, the complex **13** having *para*-xylyl spacer was found the most active (IC_50_ 9.7 μM) that verifies our previously reported results.

**Conclusions:**

All the bis-benzimidazolium salts (**8-10**) were found inactive whereas after bonding with silver cations, the Ag(I)-NHC complexes (**11-13**) showed a dose dependent cytotoxic activity. This proved that silver practice an important role in death of cancer cells. Also, the *N*-alkyl/aryl substitutions and ortho/metal/para xylyl units regulate the cytotoxicity.

## Background

Deaths due to cancer are on increase. According to global cancer statistics in 2002, there were 10.9 million new cancer cases and 6.7 million deaths [1] worldwide, whereas in 2008 this number increased to 12.66 million new cases and more than 7.5 million deaths. New drugs to fight cancer are constantly needed.

Different therapeutic options like chemotherapy, radiotherapy and surgery are now extensively used to treat various types of cancer. In chemotherapy, the discovery of cisplatin by Rosenberg and co-workers is perhaps the first historical example of anticancer metal-based drugs [2]. However, the severe side effects confined its applications. Later on, a number of its derivatives were synthesized and biologically evaluated but only few of them proved to have pharmacological advantages relative to cisplatin [3,4]. Platinum based drugs are now so frequently used that only oxaliplatin, a platinum based drug, is expected to cross 2 billion Euros business in the next two years [5]. This drug was recently approved to be used as a second line therapy in metastatic colorectal cancer and showed minor side affects so far which can be treated symptomatically. In addition to platinum based drugs, a wide range of transition-metal drugs have been at various stages of development [4,6-8], however, none of them could pass all the stages of clinical development until today [4].

Recently, Metal *N*-heterocyclic carbene (M-NHC) complexes appeared as an emerging field of research in medicinal chemistry. This is also evident by recent research reports [9-11] and several reviews [4,12-14]. It is important to highlight that a major portion of these findings is based on imidazole nucleus; however, its benzimidazole derivative has been rarely explored in organometallic chemistry [15], especially for biological applications [16]; whereas in organic chemistry a number of its organic derivatives have shown many promising therapeutic activities including cancer [17,18]. The structural resemblance of benzimidazole derivatives with the naturally occurring nucleotides allows them to interact with the biopolymers of the living system and hence, signifies their biological importance [17].

Due to aforementioned reasons, we used this chemical entity (benzimidazole) to synthesize organometallic compounds to be tested against cancer. However, while dealing with biological systems, the selection of metals is also an important and judicious issue. Silver salts have historically been used to maintain human health. Many early civilizations used silver salts for purification of drinking water, healing wound, and prevention of eye infections in newborns [14,19-21]. Worldwide extensive use of silver sulfadiazine as a tropical medicament for infected burns renders a notion on safety of silver salts. Now a days, silver is also used in consumer products like clothing, antibacterial sprays, cosmetics, cell phones, laptop keyboards, dietary supplements and more [22]. It is however, the low toxicity of silver salts for humans that has attracted researchers to further explore their biomedical applications, specifically antimicrobial and anticancer [4,14,20,23,24].

It was certain that the chemical combination of these biologically important moieties would provide a medically advanced class of compounds. Interestingly, only bis-benzimidazolium salts, the precursors to form Ag-NHC, showed potential anticancer activity [25]. However, later on we reported that silver in bonding with these bis-benzimidazolium salts through Ag-NHC route, enhances the anticancer potential [26-29]. On the other hand, our published [30] and unpublished data reflects that imidazole based ligands with similar structure, bis-imidazolium salts, are either completely inactive or have mild cytotoxicity against cancer cells. However, the imidazole based Ag(I)-NHC complexes were found to have a diverse anticancer potential.

Inspiring from our previous research on anticancer potential of benzimidazole based ligands and respective dinuclear Ag(I)-NHC complexes [26-28], in this research, *N*-^*i*^propyl substituted *ortho*/*meta*/*para* xylyl linked bis-benzimidazolium salts were synthesized either with bromide or hexaflourophosphate counter anions and were further bonded with silver metal ions in order to test their potential against cancer cells. Unlike our previous reports [26-28,31], all the ligands were found inactive but respective silver complexes showed cytotoxicity in a dose dependent manner. All of our previous and current results indicate that silver metal ions practice a role in the death of cancer cells.

## Results and discussions

### Syntheses

**Scheme 1:**
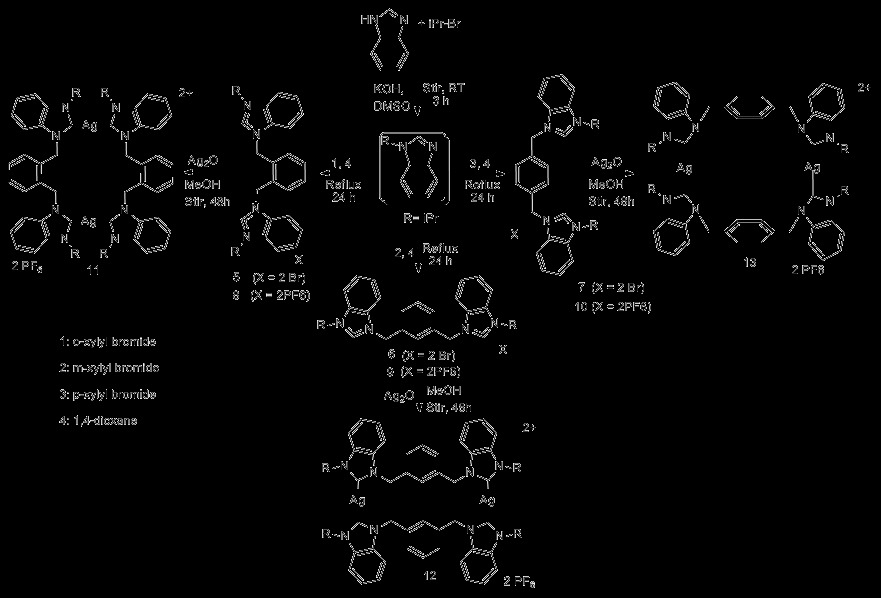
**Synthesis of *N***-^***i***^**propyl benzimidazole, *Bis*-imidazolium salts (5-10), and respective dinuclear Ag(I)-NHC complexes (11-13).**

*N*-^*i*^propylbenzimidazole was synthesized by a developed method [32] with minor modifications (see Additional file 1: Supplementary I). Furthermore the reaction of two equivalents of *N*-ipropyl benzimidazole with 1,2/1,3/1,4-(bromomethylene)benzene in 1,4-dioxane at 80-100°C for 24 h afforded the product in good yields (Scheme 1).

*In the case* when product settled as a sticky brownish fluid at the bottom of flask, the upper layer was decanted and product was washed with fresh 1,4-dioxane (3 × 5ml). The resulting bromide salt was converted directly to a hexaflourophosphate counterpart by metathesis reaction using KPF_6_ (2 equivalents) in 50 mL of methanol or H_2_O/MeOH system.The mixture was stirred for 3 h and filtered. The white precipitates were collected and washed with distilled water (2 × 5 mL), then left to dry at ambient temperature. However, *In the case* when product appeared as solid (precipitates) in the reaction medium. The precipitates were collected, washed with fresh 1,4-dioxane (3 × 5 mL), dried at RT for 24 h, and ground to fine powder (See Additional file 2: Supplementary II).

For synthesis of complexes, ligands with hexaflourophosphate counter ions were dissolved in acetonitrile alongwith Ag_2_O and stirred for a period of 2 days at room temperature. Reaction mixture was then filtered by celited 545 to collect a clear solution. In some cases filtration process had to repeat two to three times to collect a crystal clear solution. So obtained was evaprated under reduceed pressure to collect powderious material and recrystalized by acetonitrile/water system. However, ligands with bromide counter ions were dissolved in methanol following the same procedure, stopping at till filtration. So obtained was then converted to hexaflourophophate counter ion by metathesis reaction, according to which halide salt of compund is stirred at room temperature with potassium hexaflourophosphate salt. White precipitates are expected within few minute, however stirring for a period of 3 h is necessary. Precipitates were filtered by whatman filter paper, washed by water (3 × 5 mL) following by methanol (3 × 5 mL). In some cases, white precipitates do not appear and addition of water is necessary in the metathesis reaction mixture (See Additional file 3: Supplementary III).

For ligands, conversion of counter anion from halide to hexaflourophosphate is done only for the ease of handling whereas for complexes this method is the way to purity if and only if halide salt of ligand is used as starting material in methanol as describe above.

The PF_6_ salts of all the compounds are soluble in acetonitrile, DMSO, and DMF but not soluble in methanol, ethanol, and water whereas the halide salts of ligands and complexes are soluble in methanol, ethanol, DMSO, DMF and water except acetonitrile.

### FT-IR spectra of the compounds

*N*-alkyl substituted bis-benzimidazolium salts do not have many functional groups to be characterized by IR spectroscopic technique. However, it is possible to study some spectral features of these salts and their respective metal-complexes as specific patterns can be observed, which may be used as primary indicators of a successful synthesis [26].

**Figure 1:**
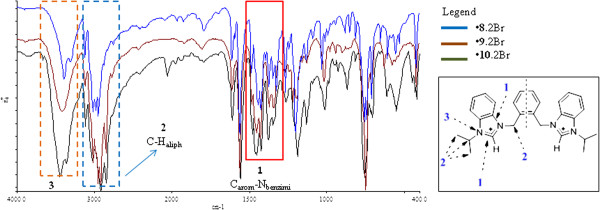
**FT-IR overly spectra of *bis*-benzimidazolium dibromide precursors (5-7. 2Br).** The peaks labeled as 3 in the red box indicate the overlap of tertiary N-atom of benzimidazolium ring by hydrated water molecules.

**Figure 2:**
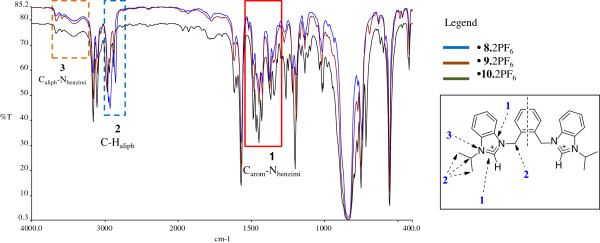
**FT-IR overly spectra of *bis*-benzimidazolium *bis *(hexaflourophosphate) precursors (8-10. 2PF**_**6**_**).** The weak FT-IR signals labeled as 3 in the red box represent the pure modes of tertiary N-atom in benzimidazolium ring.

For the halide salts of ligands (**5**-**7**.2Br), strong and sharp stretching vibrations (3402 – 3424 cm^-1^) appeared for tertiary nitrogens of azolium ring (C_aliph_-N_benzimi_) which are overlapped by water peak due to the presence of hydrated water in these compounds (see Figure 1). In single crystal structures of *bis*-benzimidazolium halides these water molecules are found to be attached with halide (X = Cl/Br) anions through X--H-O hydrogen bondings [33,34]. This type of overlapping is rare in *bis*-imidazolium halides due to absence of hydrated water molecules [35,36]. Also, the pure modes of these vibrations can be observed in PF_6_ salts of these compounds (**8-10.**2PF_6_) due to absence of hydrated water molecules (See Figure 2). The pure modes of the C-H stretching vibrational bands in *bis*-benzimidazolium salts appeared at around 2900 to 3000 cm^-1^ (C-H_aliph_). This variation in the range is due to presence of C-H (sp^3^-s) stretching of alkyl chains and methylene (N-CH_2_-Ar) group. A strong and sharp intense band was observed in the range 1350 to 1500 cm^-1^ ascribed to the stretching modes of vibrations of benzimidazole ring due to the presence of -HC=N- module [37]. It may be concluded that the reduction in the intensity of this band in benzimidazolium salts, as compare to *N*-alkylazole, is probably caused by the conjugation of C=N bond with the azole ring and due to *N*-alkylation, where alkyl group acts as electron donating entity. The other ring vibrations are intense bands at around 1050 and 1220 cm^-1^.

**Figure 3:**
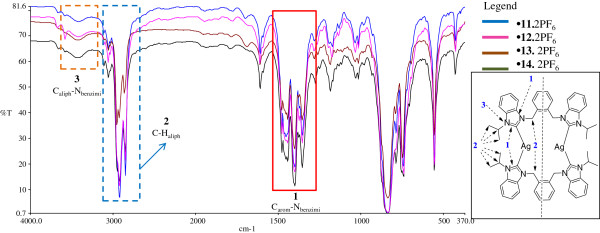
**FT-IR overly spectra of Ag(I)-NHC complexes (11-13. 2PF**_**6**_**).** The strong intense bands labeled as 1 in solid red box provided the preliminary confirmation of the formation of Ag(I)-NHC complexes.

We also found interesting features of FT-IR to confirm a successful synthesis of Ag-NHC complexes. We observed that binding of NHC carbon with silver metal ion strengthens vibrations in the range 1350 – 1500 cm^-1^ and a characteristic “*four fingers (f.fs)*” pattern appears for all the Ag-NHC complexes. This region is specific for –C=N (C_arom_-N_benzimi_) ring stretchings. The observed “*f.fs”* pattern is entirely different than all the respective vibrations in azolium salts and is easily distinguishable (See Figure 3). This is in accordance with our previous reports for similar dinuclear Ag(I)-NHC complexes [26-28].

### FT-NMR spectra of the compounds

FT-NMR spectra of all the compounds were analyzed in *d*_*6*_-DMSO, *d*_*3*_-Acetonitrile, and *d*_*4*_-methanol over the scan range 0 to 12 δ ppm for ^1^ H NMR and 0 to 210 δ ppm for ^13^ C NMR studies. In the ^1^H NMR spectra, a characteristic sharp singlet (Hd) in the range δ 10.00 – 10.30 for benzimidazolium salts for acidic proton (NCHN) indicated the successful formation of target ligands **5**-**7** &**8**-**10**, respectively [38,39]. For salts (**5-10**) the characteristic peaks for benzylic protons (N-CH_2_-Ar, Hc) and protons at secondary carbons (N-CH-R2, Hb) appeared in the range δ 5.81 – 6.40 and 4.52 – 5.20, respectively.

**Figure 4:**
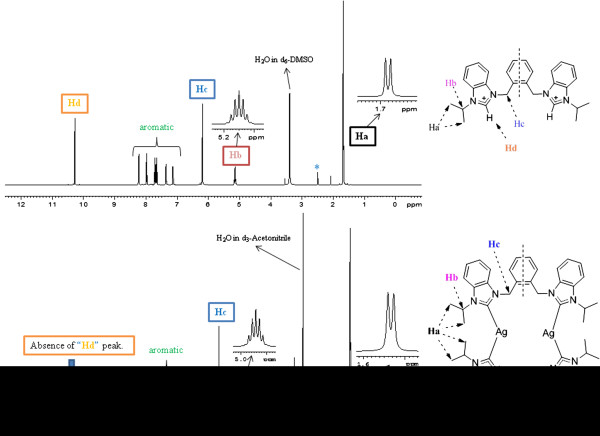
^**1**^**H NMR spectrum (*d***_***6***_-DMSO*/*d*_***3***_-acetonitrile**, 500 MHz) indicating the changes in chemical shifts after successful reaction between 5 and Ag_**2**_**O to get dinuclear complex 11.** The acidic proton signal Hd disappears on complex formation.

Synthesis of Ag-NHC complexes was confirmed by the disappearance of acidic proton peak (Hd) (See Figure 4). The signals caused by benzylic (N-CH_2_-Ar) group, which connects xylyl unit with benzimidazolium units, displayed sharp singlets in the range δ 5.13 - 5.64 ppm for silver complexes. These resonance values are comparable with corresponding ligands.

**Figure 5:**
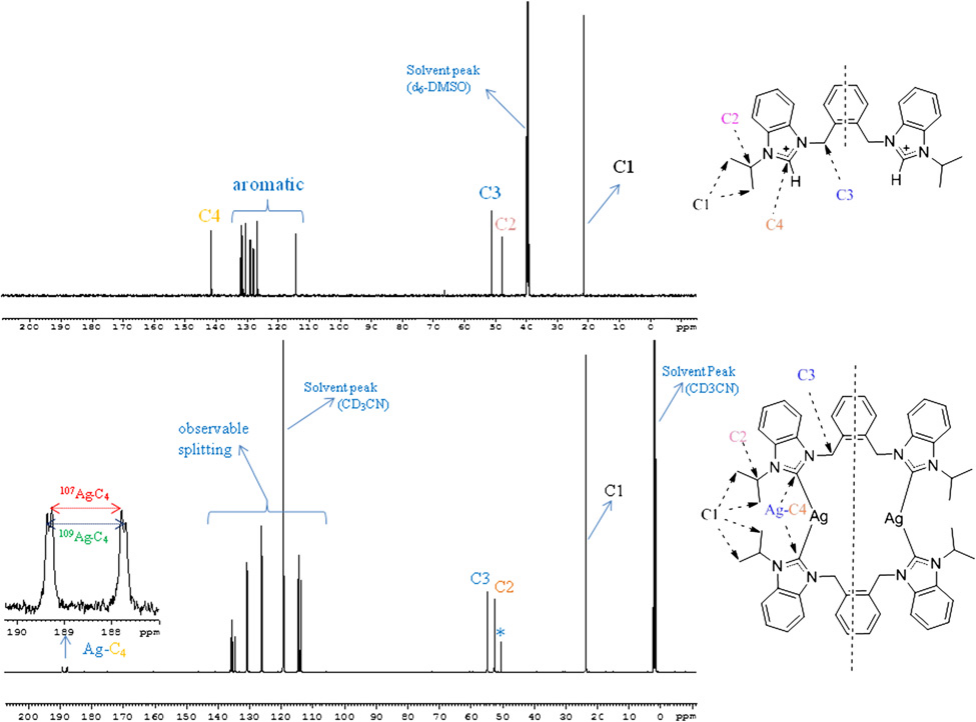
^**13**^**C NMR spectra (*d***_***6***_**-DMSO/*d***_***3***_**-acetonitrile, 125 MHz) indicating the changes in chemical shifts and appearance of two doublets after successful reaction between 5 and Ag**_**2**_**O to get dinuclear Ag-NHC complex 11. **^۩^Naturally occurring Silver (Ag) is composed of the two stable isotops ^107^Ag and ^109^Ag with ^107^Ag being the more abundant (51.84%). *J*_109Ag-C_ = 208 Hz. *J*_107Ag-C_ = 184 Hz.

Similarly, the structural features of the salts were further confirmed by the ^13^C NMR data. In ^13^C NMR spectra, the chemical shift values of C4 carbon (NCN) were observed within the range δ 141 – 143 ppm, which is also in agreement with reported data for similar azolium salts [39,40]. Upon complexation with Ag, two doublets appear at *ca*. δ 189 for benzimidazole based Ag-NHC complexes with Ag-C coupling constants *ca.* 208 Hz and 180 Hz (See Figure 5).

These doublets appear in dimeric complexes of structure [L_2_Ag_2_]^2+^ due to carbene carbon bonding to C-Ag^107^ and C-Ag^109^, respectively [26,41]. In case of imidazole based Ag-NHC complexes such doublets appear at *ca*. δ180 with splitting patterns 180 – 189 Hz for Ag^107^ and 204 – 220 Hz for Ag^109^[42]. The resonances of aromatic carbons were found in the comparable region around δ 113 – 137 in Ag(I)-NHC complexes (**11** – **13**. 2PF_6_). Also, the benzylic carbon (N-C-Ar) and alkyl chain carbon resonances were observed in the chemical shift regions δ 53.86 – 54.25 ppm and δ 23.04 – 24.79 ppm respectively. These resonance values are δ 2-3 ppm downfield as compare to corresponding ligands.

### Crystallography

The molecular structures of the benzimidazolium salts Additional files 4, 5, 6: **5**, **9, 10** were determined by single crystal X-ray diffraction studies. Crystal refinement data, selected bond lengths and angles of salts Additional files 4, 5, 6: **5**, **9, 10** are tabulated in Tables 1,2,3,4.

**Table 1:** Crystal data and structure refinement details for carbene precursor 8, 9, and 10

	**5**	**9**	**10**
Formula	C_28_H_36_N_4_Br_2_O_2_	C_28_H_32_ F_12_N_4_P_2_	C_28_H_32_ F_12_N_4_P_2_
Formula weight	620.41	714.52	714.52
Crystal system	Monoclinic	Monoclinic	Monoclinic
Space group	*P-21/c*	*P-21/c*	*P-21/n*
Unit cell dimensions			
a (Å)	10.3809(2)	16.3719(4)	12.0379(6)
b (Å)	11.7017(3)	10.0392(2)	10.2165(5)
c (Å)	11.3918(2)	19.1499(4)	12.7232(6)
α (^o^)	90.00	90.00	90.00
β (^o^)	91.742(1)	96.896(1)	96.236(1)
γ (^o^)	90.00	90.00	90.00
V (Å^3^)	1383.17(5)	3124.72(12)	1555.51(13)
*Z*	2	4	2
Density (calcd) (gm/cm^3^)	1.490	1.519	1.526
Abs coeff (mm^-1^)	2.962	0.238	0.239
F(000)	636	1464	732
Crystal size (mm)	0.13 x 0.26 x 0.35	0.23 x 0.27 x 0.39	0.10 x 0.22 x 0.27
Temperature (K)	100	100	100
Radiation (Å)	MoKa 0.71073	MoKa 0.71073	MoKa 0.71073
*θ* Min, max (^o^)	2.5, 32.7	2.1, 31.9	2.6, 33.3
Dataset	−13: 15; -17: 17; -17: 17	−20: 24; -14: 14; -28: 28	−18: 18; -15: 15; -19: 19
Tot.; Uniq. Data	20647	41315	22401
R (int)	0.026	0.033	0.026
Nref, Npar	9569, 329	10750, 419	5971, 208
R, wR_2_, S	0.0334, 0.0630, 0.93	0.0522, 0.1531, 1.04	0.0370, 0.1134, 1.04

**Table 2:** Selected bond lengths (Å) and angles (o) of 5

**C23-C24**	**1.506(3)**	**N2-C8**	**1.461(3)**	**C7-C6**	**1.394(3)**
C23-N1	1.492(2)	C8-C9	1.509(3)	C2-C7	1.391(3)
N1-C1	1.319(3)	C9-C14	1.410(3)	C3-C4	1.376(3)
C1-N2	1.343(3)	C9-C10	1.392(3)	C1-H1	0.9500
N2-C7	1.389(3)	C10-C11	1.392(3)	O1-H2 (W1)	0.8500
C24-C23-C25	111.71(18)		N2-C8-C9	114.61(16)	
C24-C23-N1	110.34(16)		C8-C9-C14	116.84(16)	
N1-C1-N2	110.32(18)		C8-C9-C10	123.94(17)	
C23-N1-C2	124.97(16)				
C1-N2-C8	124.32(18)				

**Table 3:** Selected bond lengths (Å) and angles (o) of 9

**C23-C24**	**1.520(3)**	**N2-C8**	**1.464(2)**	**C7-C6**	**1.394(2)**
C23-N1	1.486(2)	C8-C9	1.515(2)	C2-C7	1.395(2)
N1-C1	1.328(2)	C9-C14	1.397(2)	P1-F1	1.6014(13)
C1-N2	1.332(2)	C9-C10	1.392(2)	C1-H1	0.9500
N2-C7	1.395(2)	C10-C11	1.389(3)		
C23-C24-C25	111.84(15)		N2-C8-C9	111.92(13)	
C24-C23-N1	110.08(14)		C8-C9-C14	119.69(14)	
N1-C1-N2	110.91(14)		C8-C9-C10	120.69(15)	
C23-N1-C2	127.10(13)				
C1-N2-C8	126.51(13)				

**Table 4:** Selected bond lengths (Å) and angles (o) of 10

**C9-C8**	**1.5201(15)**	**N2-C2**	**1.3886(13**)	**C2-C3**	**1.3955(16)**
C8-N1	1.4864(12)	C11-C12	1.5145(14)	C4-C5	1.4020(19)
N1-C1	1.3316(12)	C12-C13	1.3957(13)	P1-F1	1.5936(9)
C1-N2	1.3349(13)	C13-C14	1.3943(14)	C1-H1	0.9500
N2-C11	1.4609(13)	C7-C6	1.3959(14)		
C9-C8-C10	112.29(9)		N2-C11-C12	112.69(8)	
C9-C8-N1	109.41(8)		C11-C12-C13	118.52(8)	
N1-C1-N2	110.64(8)		N2-C2-C3	131.12(10)	
C8-N1-C1	126.89(8)				
C1-N2-C11	125.88(8)				

**Figure 6:**
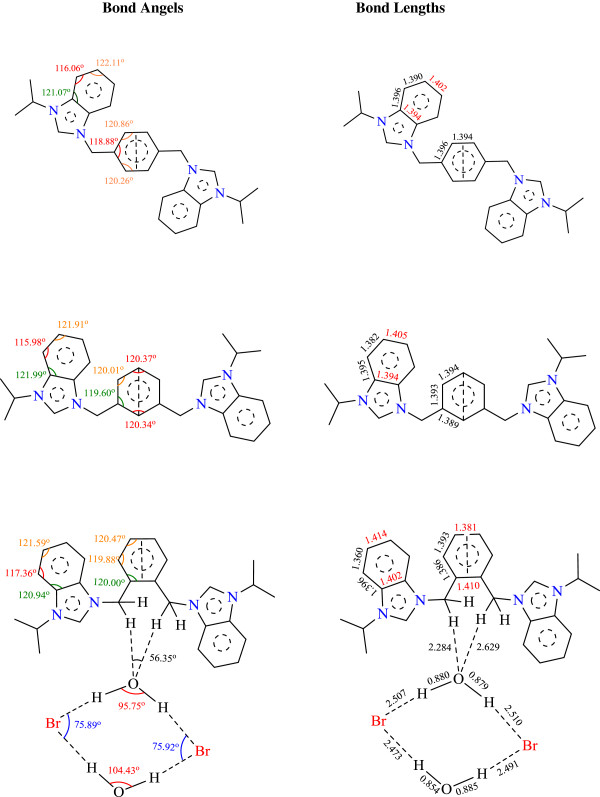
**A comparisons of bond angles and bond lengths for benzene rings of benzimidazolium and xylyl units in *bis*-benzimidazolium salts (All the bond angles and bond lengths were selected from the crystallographic data of respective salt).**

**Figure 7:**
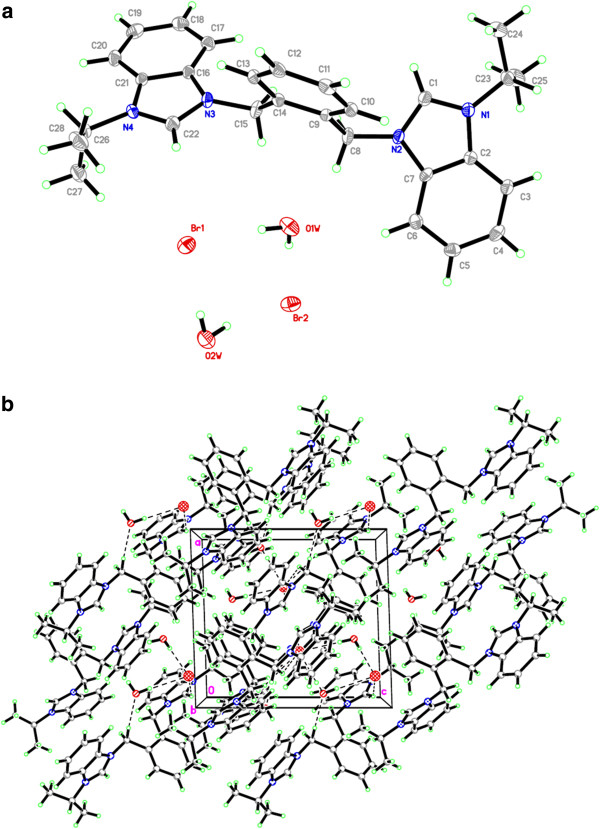
**The ORTEP picture of bis-benzimidazolium salt 5.2Br with displacement ellipsoids drawn at 50% probability and crystal packing (Monoclinic).**

Salt Additional file 4: **5** crystallizes in monoclinic space group *P21/c* with one cationic *bis*-benzimidazolium core, two bromide counter anions and two water molecules. Bromide counter anions and water molecules are connected in diamond shape formation through H-bonding. Bond angles (H-O-H) of one of the water molecules is 95.75^o^ whereas for the 2^nd^ one it is 104.43^o^. This variation in bond angels might be due to strong and weak interactions of hydrogens with bromide counter anions (See Figure 6). Crystal studies reveal that both benzimidazolium cores are almost perpendicular to xylyl unit making angles of 114.61(16)^o^ and 115.83(16)^o^ facing opposite directions. Similarly, the terminal ^*i*^propyl groups are also almost perpendicular to the planes of the benzimidazole rings making angles around 108-110^o^. These terminal ^*i*^propyl groups are also facing opposite directions on both ends of bis-benzimidazolium core with internal ^*i*^propyl angles 111.71(18) for C24-C23-C25 and 112.07(18)^o^ for C27-C26-C28. The internal imidazole ring angle (N–C–N) at the carbene center is 110.32(18)° for N1-C1-N2 and 110.30(18)° for N3-C22-N4, and these values are well within the range for similar benzimidazole based salts [34,43-45]. In the crystal, the bromide anions link the cations with a three-dimensional network via the intermolecular hydrogen bonding C-H….Br (2.869 and 2.953 Å) to benzylic hydrogens (N-CH_2_-Ar) and acidic proton (NCHN). Also water molecules are bonded with the benzimidazolium core through the intermolecular hydrogen bonding via H-O….H (2.284 and 2.629 Å) to benzylic hydrogens (N-CH2-Ar). A perspective view of the salt and its crystal packing are shown in Figure 7.

**Figure 8:**
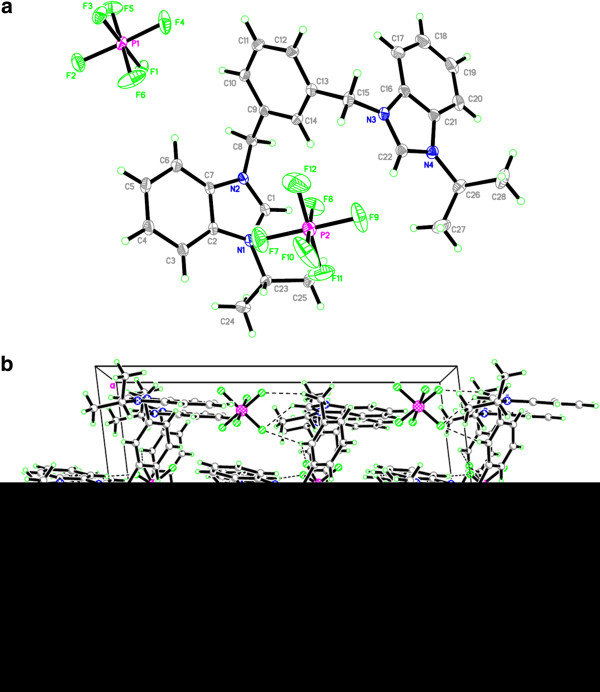
**The ORTEP picture of bis-benzimidazolium salt 9.**2PF_6_**with displacement ellipsoids drawn at 50% probability and crystal packing (Monoclinic).**

Salt Additional file 5: **9** also crystallizes in monoclinic space group *P-21/c* with one cationic bis-benzimidazolium core and two hexaflourophosphate anions. Crystal studies reveal the existence of salt in a zig-zag manner having benzimidazoles almost perpendicular to the plane of xylyl group. However, the shape of the entire molecule depends on the terminal *N-*alkyl/aryl substituent which readily offers the rigidity and finite shape. The internal imidazole ring angle (N–C–N) at the carbene center is 110.75(18)° for N4-C22-N3 and 110.91(18)° for N1-C1-N2, and these values are well within the range for similar benzimidazole based salts [34,44,45].]. The bond angles between benzimidazole ring and xylyl were found to be N2-C8-C9 = 111.92(13)^o^ and N3-C13-C15 = 112.33(13)^o^ from opposite direction. In the crystal, the hexaflourophosphate anions link the cations with a three-dimensional network via the intermolecular hydrogen bonding C-H….F (2.547 and 2.560 Å). A perspective view of the salt and its crystal packing are shown in Figure 8.

**Figure 9:**
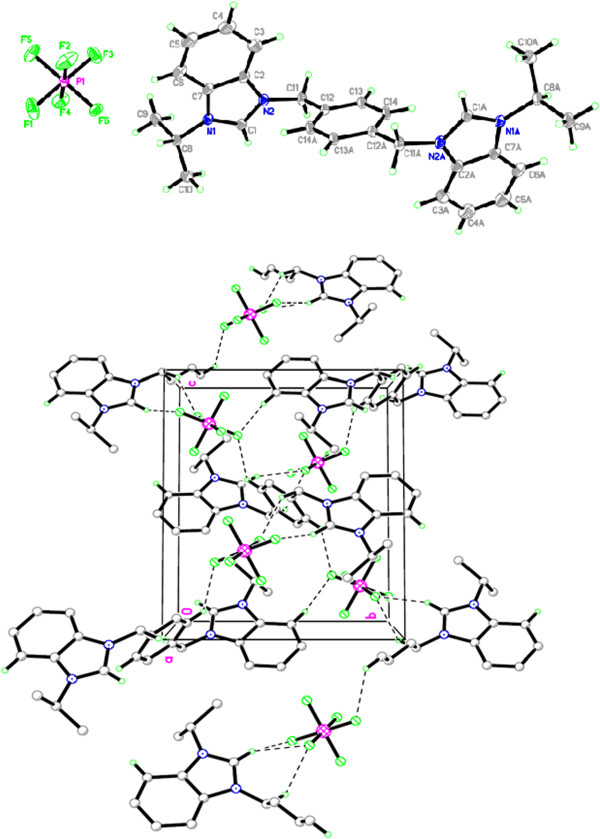
The ORTEP picture of bis-benzimidazolium salt 10.2PF_6_ with displacement ellipsoids drawn at 50% probability and crystal packing (Monoclinic).

Salt Additional file 6: **10** crystallized in monoclinic space group *P-21/n* with one cationic *para*-xylyl linked *bis*-benzimidazolium core and two hexaflourophosphate anions. Crystal studies reveal the existence of salt in a zig-zag manner having benzimidazolium units almost perpendicular to the plane of xylyl group. The internal imidazole ring angle (N–C–N) at the carbene center is 110.64(8)° for N1-C1-N2 this value is well within the range for similar benzimidazole based salts [34,44,45]. The bond angle between benzimidazolium components and xylyl group was found to be N2-C11-C12 = 112.69(8)^o^. In the crystal, the hexaflourophosphate anions link the cations with a three-dimensional network via the intermolecular hydrogen bonding C-H….F (2.421 and 2.450 Å). A perspective view of the salt and its crystal packing are shown in Figure 9.

### Benzene rings of benzimidazolium and xylyl units

Interestingly, benzene rings of benzimidazolium and xylyl units have uniquely different bond lengths and angels. This might be due to different substitutions that create different environment for each incorporated atom in aromatic system. For example, benzene ring of benzimidazolium unit is not symmetric and is little bit compressed. This might be due to electron withdrawing effect of imidazolium cation. Figure 6 highlights the bond angels 115.98^o^, 116.06^o^, & 117.36^o^ in red. These bond angels are 3^o^-5^o^ lesser than the bond angels of neighboring atoms within the same ring. However, this difference is smaller for benzene rings of xylyl unit i.e., 1^o^-2^o^. Also, the bond lengths of both types of benzenes have the same story. *This proves that two neighboring aromatic systems with comparatively different ring sizes affect the geometry of each other*.

### *In vitro* anticancer activity

In this study, antiproliferative potential of three newly synthesized bis-benzimidazolium salts, with halide & PF_6_ counteranions (**5-10**) and their Ag(I)-NHC complexes (**11-13**) were evaluated using MTT assay on human colorectal cancer cells (HCT 116). The results were reported as mean percentage inhibition of cell proliferation (±SD). All the bis-benzimidazolium salts with bromide as counteranions (**5-7**) were found to be relatively active (IC_50_ = 85, 0.2, & 157.52 μM for **5**, **6**, & 7 respectively) compare to the same benzimidazolium salts with hexaflourophosphate as counteranions (IC_50_ = > 200 μM for compounds **8-10**). This is in accordance with our previous reports [25-28] where we mentioned that halide salts of compounds with these structures are relatively active compare to the compounds with hexaflourophosphate as counter anions. The counteranions of these salts are usually exchanged from halide to PF_6_ for the ease of handling and in some cases to achieve the purity because these compounds as PF_6_ salts appear as white powder whereas in halide form these salts are mostly thick oily fluids before recystalization. The silver complexes (**11**-**13**) showed relatively better antiproliferation activity with IC_50_ (concentration of the test compound to achieve 50% inhibition) 43, 44.5, and 9.7 μM for complexes **11**, **12**, and **13** respectively.

**Figure 10:**
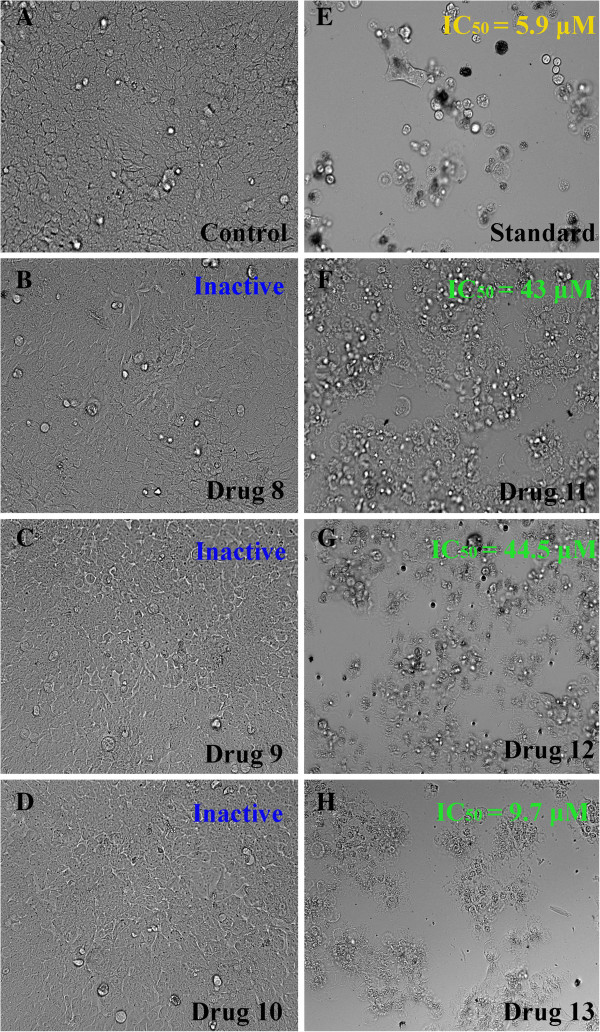
**HCT116 cell images were taken under an inverted phase-contrast microscope at × 200 magnification with a digital camera at 48 h after treatment with the samples. A**) Cells from the control group showed fully confluent growth with compactly proliferating HCT 116 cells. Treatment with **8** (**B**), **9** (**C**) and **10** (**D**) showed negligible cytotoxicity, as the cell growth did not get affected and cellular morphology was similar to that of negative control. E) Photomicrograph depicts the strong cytotoxic effect of 5-fluorouracil (IC_50_ = 5.9 μM). It can be seen clearly that the compound affected the normal morphology of all most all the cells of the group which rendered the cells to lose their viability. Except few affected cells, only the cellular debris remained can be seen in the growth medium. **F**) Treatment with **11** showed significant inhibition in HCT 116 cell proliferation with IC_50_ = 43 μM, as the population of cells reduced drastically within the 48 h of treatment. The picture revealed that, the treated cells exhibited apoptotic cellular death, which is evident through the appearance of membrane blebbing, nuclear condensation and apoptotic bodies. **G**) HCT 116 cells treated with the compound **12** exhibited considerable cytotoxicity (IC_50_ = 44.5 μM). As the photomicrograph depicts the treatment of HCT 116 cells with the compound showed significant inhibitory effect on the cellular growth as compared to the untreated cells. **H**) Cells treated with the **13** (IC_50_ = 9.7 μM) showed most potent cytotoxicity among all the tested samples. The activity is comparable to that of standard reference, 5-fluorouracil. The viability of the cells was severely affected, as the photomicrograph showed all the treated cells lost their viable characteristic features.

**Figure 11:**
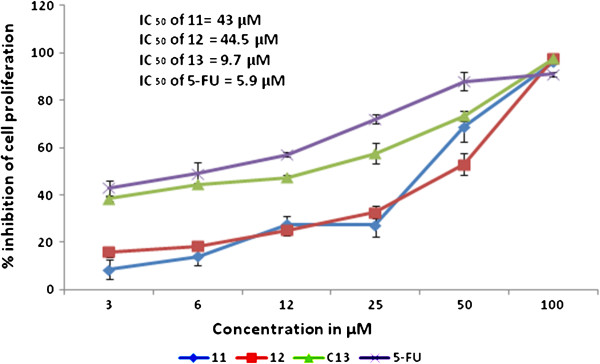
**Anti-proliferation effect of test compounds on HCT 116 was assessed by MTT-assay.** The figure depicts dose-dependent activity of test complexes (11-13). The activity of silver complexes was found significant compare to respective salts however all the synthesized drugs were less profound than 5-FU (values are represented as mean ± SD n=3).

The pure metals are inactive; however, the activity of metal cations varies on their bioavailability. Hence delivery methods, solubility, and ionization of the silver sources are significant parameters to deal metals in biological systems [46]. Possibly this is the reason that the bonding of silver cations to biologically compatible ligands enhances the bioavailability and ultimately the activity of silver cations. This is very obvious with our current and previously reported results [25,26,30,47], where ligands were found to be relatively less active compare to respective silver complexes. The photomicrograph of the cells treated with the silver complexes revealed the morphological features of apoptosis consist of membrane blebbing, nuclear condensation and formation of apoptotic bodies (Figure 10). It is reported that silver-*N*-heterocyclic carbene complexes induce caspase independent apoptosis via mitochondrial apoptosis-inducing factor [9]. Several studies showed that silver cations bind to the cell surfaces and interact with the enzymes and proteins that are important for the cell wall synthesis [19,26,46], this property of silver was confirmed in the photomicrographs of cells treated with the silver complexes where the silver deposition can be clearly visualized in the membrane as well as around the cellular organelles (Figure 10). The variation in *N*-alkyl chain length might further affect the potency of these compounds as the substituent produces variation in the lipophilicity of the drugs [26,28]. Thus, the antiproliferative effect of tested complexes is likely due to the lipophilicity of the complexes that alleviates the transport of silver complexes into the cell and posteriorly into the organelles where silver may possibly contribute to toxicity by inhibiting cellular respiration and metabolism of biomolecules. Figure 11 shows the dose dependent antiproliferative effect of synthetic complexes (**11**-**13**) whereas the ligands (**8-10**) having PF_6_ counteranions were found inactive and the ligands (**5-7**) having bromide counteranions showed mild cytotoxicity; hence dose dependent charts for these compounds were not included.

From our previous studies we have noticed an interesting phenomenon with respect to the n-alkyl *N*-substitutions in benzimidazole ring where the activity of silver complexes increased with increasing the chain length [26,28]. However, in the present work the experimental data for iso-propyl substituted compounds reflects that the compounds are not as potential as with n-alkyl substitutions. Also, recently we reported imidazole based similar ortho-, meta-, and para-xylyl linked salts and respective Ag(1)-NHC complexes with *in vitro* anticancer activity against the same cell line (HCT 116), where we found that the allyl-substituted para-xylyl linked dinuclear Ag(I)-NHC complex the demonstrated higher cytotoxicity with IC_50_ 0.9 μM [30]. Consistently, in the present work the benzimidazole based para-xylyl linked iso-propyl-substituted complex (**13**) demonstrated higher cytotoxicity with IC_50_ 9.7 μM, whereas the ortho- as well as meta- xylyl linked complexes (**11** and **12**) demonstrated comparatively moderate activities. This activity could be attributed to the cavity and size of the molecule. The molecules with para substitution assume bigger cavity and larger size compared to the ortho and meta units which increases the exposure of *N*-heterocyclic carbene (NHC) rings. Moreover, from the present work it is observed that the isopropyl substitution did not show improved activity than compared to the n-alkyl substitution. The most probable reason could be the symmetry of the molecule. The more symmetric the molecule is the lesser the cytotoxicity [29]. The results showed that the chain length regulates the symmetry of the molecule, the longer the chain length, the lesser the symmetry. Further studies are under investigation to understand the differential efficacies of the compounds.

## Conclusions

In conclusion, Ag(I)-NHC complexes were found relatively active compared to the respective ligands indicating that silver cations play a vital role against cancer cells. The higher cytotoxic effect of Ag(I)-NHC complexes might be due to the deposition of silver cations in the membrane of cells and the organelles and thus, possibly causing the toxicity by interfering with cellular respiration and metabolism of biomolecules. The microscopic results revealed that, the toxicity induced by the silver complexes might be via apoptosis pathway, as induction of membrane blebbing, chromatin condensation and formation of apoptotic bodies was observed. The findings of the present work conclude that the para-xylyl linked compounds are more active compared to the complexes with ortho- and meta- compartments. In addition, the iso-propyl *N*-substituted compounds are not as potential as with n-alkyl/allyl *N*-substituted compounds having similar structure; since the chain length regulates the symmetry and lipophilicity of these compounds.

## Experimental

### Reagents and instruments

Nuclear magnetic resonance spectra were recorded on Bruker 500 MHz Ultrashield™ spectrometers at ambient temperature. ^1^H and ^13^C NMR peaks are labelled as singlet (s), doublet (d), triplet (t), quartet (q), pentet (pent), sextet (sext), septet (sept) and multiplet (m), Chemical shifts were referenced with respect to solvent signals. Elemental analysis was carried out on a Perkin Elmer series II, 2400 microanalyzer. X-ray diffraction data were taken with Bruker SMART APEX2 CCD area-detector diffractometer available. The melting and boiling points were assessed by using a Stuart Scientific SMP-1 (UK) instrument. Chemicals and solvents were used as received without further purifications.

RPMI 1640 was purchased from ScienCell, USA. Trypsin and heat inactivated foetal bovine serum (HIFBS) were obtained from GIBCO, UK. Phosphate buffered saline (PBS), penicillin/streptomycin (PS) solution, MTT reagent and 5- fluorouracil were purchased from Sigma-Aldrich, Germany. All other chemicals used in this study were analytical grade or better.

## Syntheses

### Synthesis of *N*-substituted *bis*-benzimidazolium salts (5-10)

#### Synthesis of 3,3'-[(1,2-phenylenebis(methylene)]bis(1-^i^propyl-benzimidazolium) bis-hexaflourophosphate(5/8)

*N*-^*i*^propyl benzimidazole (3.20 g, 0.02 mol) was added drop wise in a vigorously stirring solution of 1,2-dibromomethyl benzene (2.36 g, 0.01 mol) in 40 ml of 1,4-dioxane and refluxed for 24 h. The product appeared as powderous material in reaction medium, filtered, washed by fresh dioxane (3 × 5 mL), and dried at room temperature for 24 h. General reaction involved in the preparation of *N*-alkyl substituted *bis*-benzimidazolium salts is shown in Scheme 1. Single crystals suitable for x-ray diffraction study were obtained by slow evaporation of saturated solution of title compound in methanol/water (3:1) at room temperature.

Colorless plates. Yield 5.13g (88.14%), mp: 266-268°C. ^**1**^**H NMR** (500 MHz, *d*_*6*_-DMSO): 1.68 (12H, d, 4 × CH_3_, *J* = 6.5 Hz), 5.15 (2H, spet., 2 × N-CH), 6.19 (4H, s, 2 × N-CH_2_-Ar), 7.13 (2H, q, Ar 2 × CH, *J* = 3.5 Hz), 7.36 (2H, q, Ar 2 × CH, *J* = 3.5 Hz), 7.65 (2H, t, Ar 2 × CH, *J* = 7.5 Hz), 7.71 (2H, t, Ar 2 × CH, *J* = 7.5 Hz), 7.98 (2H, d, Ar 2 × CH, *J* = 8.5 Hz), 8.24 (2H, d, Ar 2 × CH, *J* = 8.0 Hz), 10.28 (2H, s, 2 × NCHN); ^**13**^**C{**^**1**^**H}NMR(**125 MHz, *d*_*6*_-DMSO): 21.4 (2 × CH_3_), 47.7 (2 × N-CH), 51.0 (Ar-CH_2_-N), 114.3 (Ar-C, d, *J* = 13.7 Hz), 126.7 (Ar-C, d, *J* = 6.2 Hz), 127.9, 128.9, 130.6, 131.3, 132.0 (Ar-C) and 141.5 (NCHN). **FT-IR** (KBr): υ (cm^-1^); 3479, 3423, 3377 (C_aliph_-N_benzimi_); 3242, 3125 (C-H_arom_); 3033, 2972 (C-H_aliph_), 1625, 1607, 1559 (C_arom_-C_arom_); 1481, 1457, 1430, 1396, 1374 (C_arom_-N_benzimi_). Anal. Cal. For: C_28_H_32_Br_2_N_4_: C, 57.55; H, 5.52; N, 9.95%. Found: C, 58.10; H, 5.66; N, 9.80%. Analytical and spectral results of **8**.PF_6_ salt were found comparable with its structure.

#### Synthesis of 3,3'-[(1,3-phenylenebis(methylene)]bis(1-^i^propyl-benzimidazolium) bis-hexaflourophosphate (6/9)

Compound **6** was prepared according to the same procedure for **5**; *N*-^*i*^propyl benzimidazole (3.20 g, 0.02 mol) and 1,4-dibromomethyl benzene (2.36 g, 0.01 mol). Single crystals suitable for x-ray diffraction study were obtained by slow evaporation of saturated solution of title compound in methanol/water (3:1) at room temperature.

White powder. Yield 3.81 g (65.46%), mp: 118-120ºC. ^**1**^**H NMR** (500 MHz, *d*_*4*_-methanol): 0.46 (12H, d, 4 × CH_3_, *J* = 7.0 Hz), 3.79 (2H, spet., 2 × N-CH), 4.52 (4H, s, 2 × N-CH_2_-Ar), 6.17-6.23 (3H, m, Ar 3 × CH), 6.28 (1H, d, Ar 1 × CH, *J* = 7.5 Hz), 6.36 (2H, t, Ar 2 × CH, *J* = 8.0 Hz), 6.42 (1H, d, Ar 1 × CH, *J* = 8.5 Hz), 6.51 (1H, s, Ar 1 × CH), 6.74 (2H, d, Ar 2 × CH, *J* = 8.5 Hz), 8.69 (2H, s, 2 × NCHN); ^**13**^**C{**^**1**^**H} NMR (**125 MHz, *d*_*4*_-methanol): 22.4 (2 × CH_3_), 51.8 (2 × N-CH), 53.1 (Ar-CH_2_-N), 115.3 (Ar-C, d, *J* = 11.2 Hz), 128.4 (Ar-C, d, *J* = 8.7 Hz), 129.4, 130.2, 131.4, 132.9, 136.2 (Ar-C) and 141.7 (NCHN). **FT-IR** (KBr): υ (cm^-1^); 3498, 3398 (C_aliph_-N_benzimi_); 3160, 3060 (C-H_arom_); 2982, 2938 (C-H_aliph_), 1654, 1610, 1563 (C_arom_-C_arom_); 1478, 1431, 1494, 1394, 1340 (C_arom_-N_benzimi_ Anal. Cal. For: C_28_H_32_Br_2_N_4_: C, 57.55; H, 5.52; N, 9.95%. Found: C, 58.10; H, 5.66; N, 9.80%. Analytical and spectral results of **9**.PF_6_ salt were found comparable with its structure.

#### Synthesis of 3,3'-[(1,4-phenylenebis(methylene)]bis(1-^i^propyl-benzimidazolium) bis- hexaflourophosphate (7/10)

Compound **7** was prepared according to the same procedure for **5**; *N*-^*i*^propyl benzimidazole (3.20 g, 0.02 mol) and 1,4-dibromomethyl benzene (2.36 g, 0.01 mol).

White crystalline powder. Yield 4.92 g (84.53%), mp: 262-264^o^C. ^**1**^**H NMR** (500 MHz, *d*_*4*_-methanol): 1.80 (12H, d, 4 × CH_3_, *J* = 6.5 Hz), 5.15 (2H, spet., 2 × N-CH), 5.86 (4H, s, 2 × N-CH_2_-Ar), 7.62-7.65 (6H, t, Ar-CH), 7.71 (2H, t, Ar 2 × CH, *J* = 8.5 Hz), 7.85 (2H, d, Ar 2 × CH, *J* = 8.5 Hz), 8.09 (2H, d, Ar 2 × CH, *J* = 8.0 Hz), 10.00 (2H, s, 2 × NCHN); ^**13**^**C{**^**1**^**H} NMR (**125 MHz, *d*_*4*_-methanol): 22.3 (2 × CH_3_), 51.6 (2 × N-CH), 53.0 (Ar-CH_2_-N), 115.2 (Ar-C, d, *J* = 13.7 Hz), 128.4 (Ar-C, d, *J* = 12.5 Hz), 130.2, 132.6, 132.9, 135.9 (Ar-C) and 141.5 (NCHN). **FT-IR** (KBr): υ (cm^-1^); 3441, 3388 (C_aliph_-N_benzimi_); 3118, 3019 (C-H_arom_); 2974, 2928 (C-H_aliph_); 1613, 1557 (C-H_aliph_); 1487, 1427, 1373, 1318 (C_arom_-N_benzimi_ Anal. Cal. For: C_28_H_32_Br_2_N_4_: C, 57.55; H, 5.52; N, 9.95%. Found: C, 57.63; H, 5.66; N, 9.80%. Analytical and spectral results of **10**.PF_6_ salt were found comparable with its structure.

### Synthesis of Ag-NHC Complexes (11-13)

#### Synthesis of 3,3'-[1,2-phenylenebis(methylene)]bis(1-^i^propyl-benzimidazolium) disilver (I) bis(hexaflourophosphate) (11)

Compound **5** (1.68 g, 0.002 mol) was dissolved in methanol (100 mL) alongwith Ag_2_O (0.7 g, 0.003 mol) with excluion of light by enveloping flask with aliminum foil. The reaction mixture was stirred for a period of 2 days at room temperature, filtered by celites 545, to collect a crystal clear solution. So obtained was stirred with potassium hexaflourophosphate (0.87 g, 0.003 mol). Title compound appeared as white powder in methaol, filtered by whatman filter paper, washed by distilled water (3 × 5 mL) following by methanol (3 × 5 mL). Dried under vaccum for 24 h. Synthesis of benzimidazole based Ag-NHC complexes is shown in Sheme 2.

White powder. Yield 1.10g (81.48%), mp 240-242^o^C. ^**1**^**H NMR** (500 MHz, *d*_*3*_-acetonitrile): 1.54 (24H, d, 8 × CH_3_, *J* = 7.0 Hz), 4.94 (4H, spet., 2 × N-CH), 5.64 (8H, s, 2 × N-CH_2_-Ar), 7.10 (4H, q, Ar 4 × CH, *J* = 3.5 Hz), 7.26 (4H, q, Ar 2 × CH, *J* = 3.5 Hz), 7.39 (4H, t, Ar 4 × CH, *J* = 8.0 Hz), 7.45-7.51 (8H, m, Ar 8 × CH), 7.83 (4H, d, Ar 2 × CH, *J* = 8.0 Hz); ^**13**^**C{**^**1**^**H} NMR (**125 MHz, *d*_*3*_-acetonitrile): 23.0 (4 × CH_3_), 51.9 (4 × N-CH), 54.2 (Ar-CH_2_-N), 114.0 (Ar-C, d, *J* = 85.0 Hz), 125.6 (Ar-C, d, *J* = 22.5 Hz), 130.3 (Ar-C, d, *J* = 18.7 Hz), 134.0, 124.8 (Ar-C) and 188 [d, ^1^*J*_(C-109Ag)_ = 208 Hz] & [d, ^1^*J*_(C-107Ag)_ = 184 Hz]. **FT-IR** (KBr): υ (cm^-1^); 3426 (C_aliph_-N_benzimi_); 3156, 3060 (C-H_arom_); 2979, 2939, 2880 (C-H_aliph_), 1607, 1560 (C_arom_-C_arom_); 1477, 1442, 1411, 1393, 1373, 1341 (C_arom_-N_benzimi_). Anal. Cal. For: C_56_H_60_Ag_2_F_12_N_8_P_2_: C, 49.79; H, 4.48; N, 8.30%. Found: C, 49.50; H, 4.66; N, 8.45%.

#### Synthesis of 3,3'-[1,3-phenylenebis(methylene)]bis(1-^i^propyl-benzimidazolium) disilver (I) bis(hexaflourophosphate) (12)

Compound **12** was synthesized by following the same procedure for **8**; Compound **6** (1.23 g, 0.002 mol) and Ag_2_O (0.7 g, 0.003 mol).

White powder. Yield 0.87g (64.44%), mp: 242-244°C. ^**1**^**H NMR** (500 MHz, *d*_*6*_-DMSO): 0.48 (24H, d, 8 × CH_3_, *J* = 7.0 Hz), 3.52 (4H, spet., 4 × N-CH), 5.13 (8H, s, 4 × N-CH_2_-Ar), 6.17-6.23 (6H, m, Ar 3 × CH), 6.59 (2H, d, Ar 1 × CH, *J* = 7.5 Hz), 6.39 (4H, t, Ar 2 × CH, *J* = 8.5 Hz), 6.44 (2H, d, Ar 1 × CH, *J* = 8.5 Hz), 6.55 (1H, s, Ar 1 × CH), 6.78 (4H, d, Ar 2 × CH, *J* = 8.5 Hz); ^**13**^**C{**^**1**^**H} NMR** (125 MHz, *d*_*6*_-DMSO): 24.7 (4 × CH_3_), 52.7 (4 × N-CH), 54.2 (Ar-CH_2_-N), 115.3 (Ar-C, d, *J* = 13.7 Hz), 128.3 (Ar-C, d, *J* = 8.7 Hz), 129.6, 130.5, 131.7, 132.6, 136.2 (Ar-C) and 188 [d, ^1^*J*_(C-109Ag)_ = 208 Hz] & [d, ^1^*J*_(C-107Ag)_ = 181 Hz]. **FT-IR** (KBr): υ (cm^-1^); 3396 (C_aliph_-N_benzimi_); 3119, 3031 (C-H_arom_); 2979, 2929 (C-H_aliph_), 1654, 1610, 1563 (C_arom_-C_arom_); 1478, 1431, 1394, 1340 (C_arom_-N_benzimi_ Anal. Cal. For: C_56_H_60_Ag_2_F_12_N_8_P_2_: C, 49.79; H, 4.48; N, 8.30%. Found: C, 49.55; H, 4.64; N, 8.47%.

#### Synthesis of 3,3'-[1,4-phenylenebis(methylene)]bis(1-^i^propyl-benzimidazolium) disilver (I) bis(hexaflourophosphate) (13)

Compound **13** was synthesized by following the same procedure for **8**; Compound **7** (1.23 g, 0.002 mol) and Ag_2_O (0.7 g, 0.003 mol).

Beige colored powder. Yield 1.18 g (87.04%), mp: 320-322^o^C. ^**1**^**H NMR** (500 MHz, *d*_*6*_-DMSO): 1.80 (24H, d, 8 × CH_3_, *J* = 7.0 Hz), 5.42 (4H, spet., 4 × N-CH), 5.60 (8H, s, 4 × N-CH_2_-Ar), 7.11 (8H, s, Ar-CH), 7.32 (6H, t, Ar-CH), 7.44 (4H, m, Ar-CH), 7.80 (4H, d, Ar 4 × CH, *J* = 8.0 Hz); ^**13**^**C{**^**1**^**H} NMR** (125 MHz, *d*_*6*_-DMSO): 23.3 (4 × CH_3_), 53.4 (4 × N-CH), 53.8 (Ar-CH_2_-N), 113.8 (Ar-C, d, *J* = 92.5 Hz), 125.4 (Ar-C, d, *J* = 22.5 Hz), 128.2, 134.0, 134.8, 137.0 (Ar-C) and 189 [d, ^1^*J*_(C-109Ag)_ = 209 Hz] & [d, ^1^*J*_(C-107Ag)_ = 183 Hz]. **FT-IR** (KBr): υ (cm^-1^); 3430 (C_aliph_-N_benzimi_); 3109, 3060 (C-H_arom_); 2976, 2933, 2881 (C-H_aliph_); 1611, 1518 (C-H_aliph_); 1477, 1439, 1424, 1395, 1355, 1341 (C_arom_-N_benzimi_). Anal. Cal. For: C_56_H_60_Ag_2_F_12_N_8_P_2_: C, 49.79; H, 4.48; N, 8.30%. Found: C, 49.63; H, 4.68; N, 8.39%.

### *In vitro* anticancer activity

#### Preparation of cell culture

Initially, HCT 116 cells were allowed to grow under optimal incubator conditions. Cells that had reached a confluence of 70-80% were chosen for cell plating purposes. Old medium was aspirated out of the plate. Next, cells were washed using sterile phosphate buffered saline (PBS) (pH 7.4), 2-3 times. PBS was completely discarded after washing. Following this, trypsin was added and distributed evenly onto cell surfaces. Cells were incubated at 37°C in 5% CO_2_ for 1 min. Then, the flasks containing the cells were gently tapped to aid cells segregation and observed under inverted microscope (if cells segregation is not satisfying, the cells will be incubated for another minute) Trypsin activity was inhibited by adding 5 ml of fresh complete media (10% FBS). Cells were counted and diluted to get a final concentration of 2.5 × 10^5^ cells/mL, and inoculated into wells (100 μL cells/well). Finally, plates containing the cells were incubated at 37°C with an internal atmosphere of 5% CO_2_.

#### MTT assay

Cancer cells (100 μL cells/well, 1.5 × 10^5^ cells/mL) were inoculated in wells of microtitre plate. Then the plate was incubated overnight in CO_2_ incubator in order to allow the cell for attachment. 100 μL of test substance were added into each well containing the cells. Test substance was diluted with media into the desired concentrations from the stock. The plates were incubated at 37°C with an internal atmosphere of 5% CO_2_. After 72 h treatment period, 20 μL of MTT reagent was added into each well and incubated again for 4 h. After this incubation period, 50 μL of MTT lysis solution (DMSO) was added into each well. The plates were further incubated for 5 min in CO_2_ incubator. Finally, plates were read at 570 and 620 nm wavelengths using a standard ELISA microplate reader. Data were recorded and analyzed for the assessment of the effects of test substance on cell viability and growth inhibition. The percentage of growth inhibition was calculated from the optical density (OD) that was obtained from MTT assay. 5-FU was used as the standard reference drug.

## Competing interests

The authors declare that they have no competing interests.

## Authors' contributions

RAH supervised the project. MAI designed, synthesized, and characterized all the compounds. AMSAM and MBKA supervised the anticancer activity whereas the activity was conducted by MAI, SFN, and EF. The article was prepared and communicated by MAI. All authors read and approved the final manuscript.

## Supplementary Material

Additional file 1 Supplementary I. Click here for file

Additional file 2 Supplementary II. Click here for file

Additional file 3 Supplementary III. Click here for file

Additional file 4 CIF file of salt 5. Click here for file

Additional file 5 CIF file of salt 9. Click here for file

Additional file 6 CIF file of salt 10. Click here for file
