# Supplementary figures and images for: Potential of silver against human colon cancer: (synthesis, characterization and crystal structures of xylyl (Ortho, meta, &Para) linked bis-benzimidazolium salts and Ag(I)-NHC complexes: In vitro anticancer studies)
Source: Chem Cent J. 2013 Feb 7;7:27. doi: 10.1186/1752-153X-7-27 (PMC3637141; doi:10.1186/1752-153X-7-27)

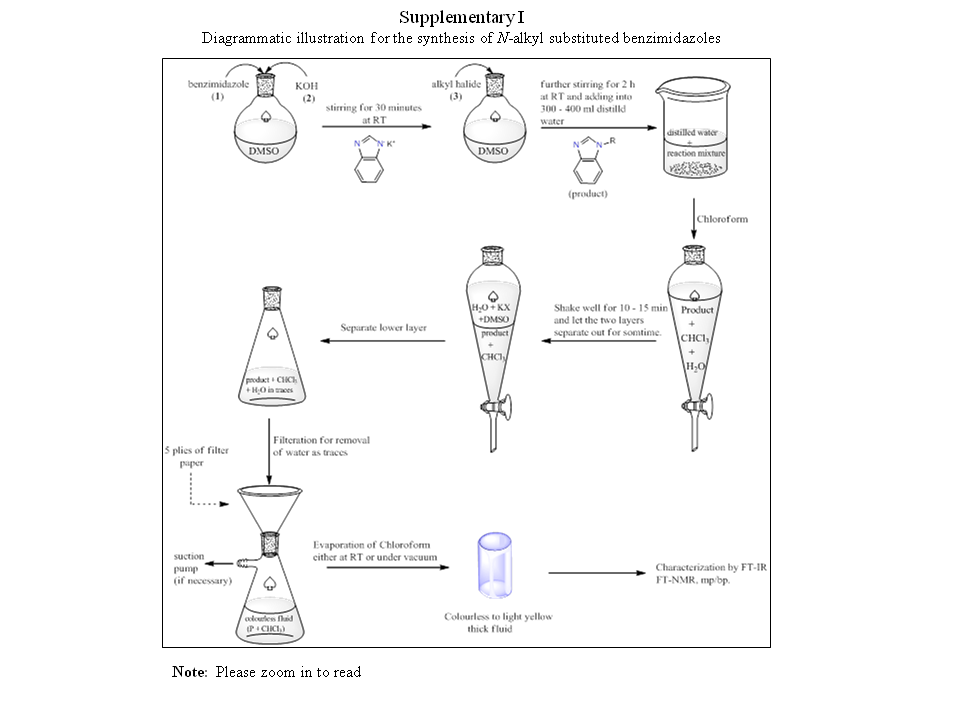

Supplement: Additional file 1 — Supplementary I. [file 1752-153X-7-27-S1.tiff]

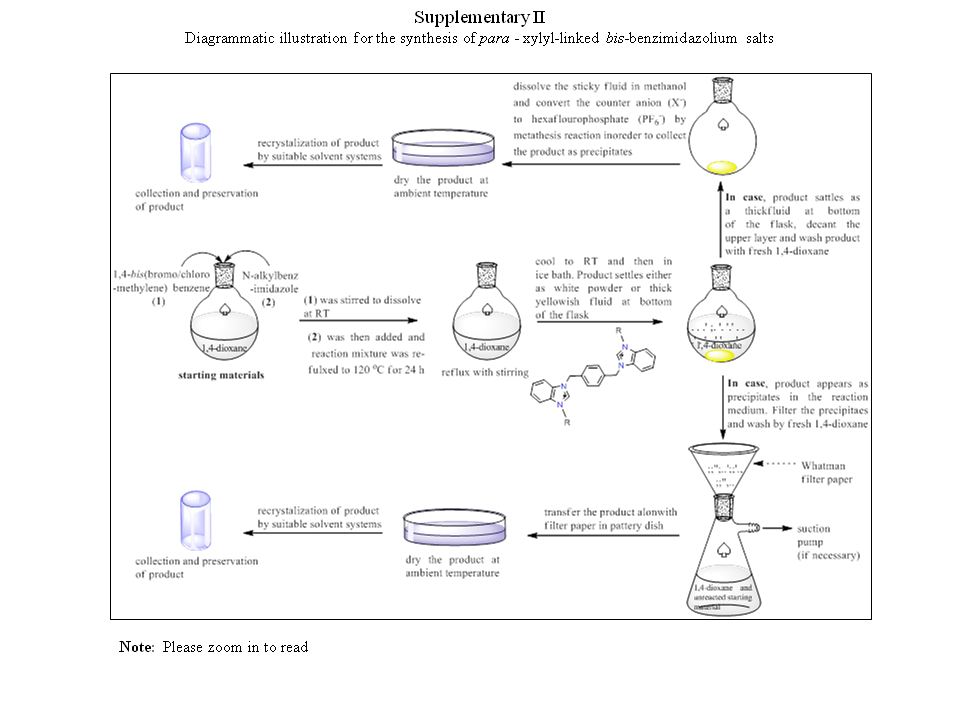

Supplement: Additional file 2 — Supplementary II. [file 1752-153X-7-27-S2.tiff]

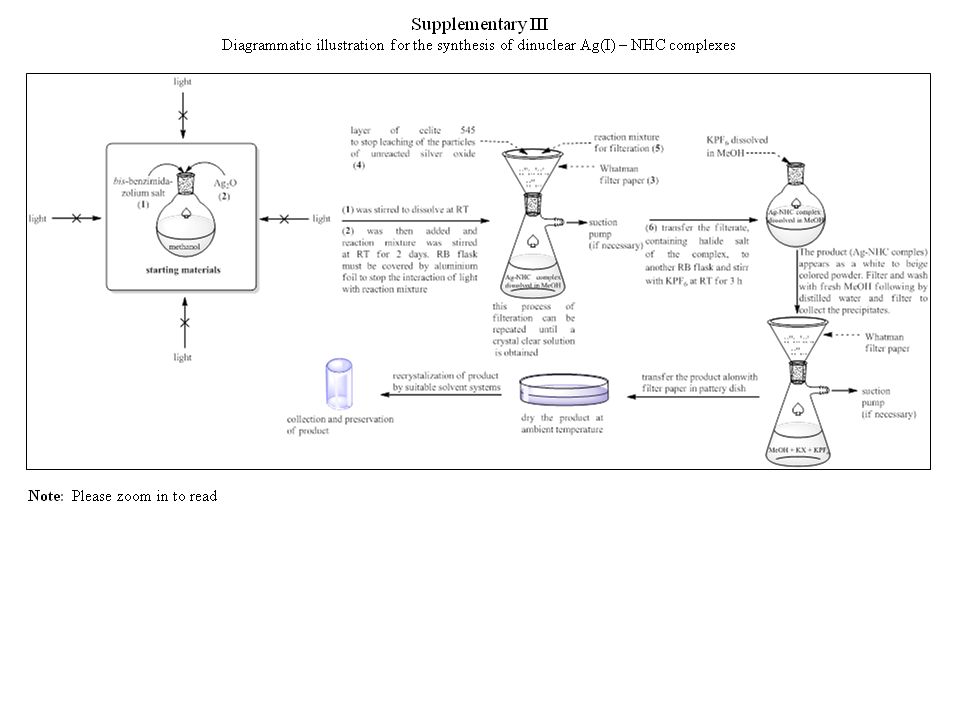

Supplement: Additional file 3 — Supplementary III. [file 1752-153X-7-27-S3.tiff]
